# The effect of yoga on sleep quality and insomnia in women with sleep problems: a systematic review and meta-analysis

**DOI:** 10.1186/s12888-020-02566-4

**Published:** 2020-05-01

**Authors:** Wei-Li Wang, Kuang-Huei Chen, Ying-Chieh Pan, Szu-Nian Yang, Yuan-Yu Chan

**Affiliations:** 1 Department of Psychiatry, Taoyuan Armed Forces General Hospital, Taoyuan, Taiwan; 2 Department of Psychiatry, Beitou Branch, Tri-Service General Hospital, National Defense Medical Center, Taipei, Taiwan; 3 Department of Psychology, Chung Yuan Christian University, Taoyuan, Taiwan

**Keywords:** Yoga, Sleep quality, Insomnia, Women, Complementary and alternative medicine, Meta-analysis, Review

## Abstract

**Background:**

To examine the effectiveness and safety of yoga of women with sleep problems by performing a systematic review and meta-analysis.

**Methods:**

Medline/PubMed, ClinicalKey, ScienceDirect, Embase, PsycINFO, and the Cochrane Library were searched throughout the month of June, 2019. Randomized controlled trials comparing yoga groups with control groups in women with sleep problems were included. Two reviewers independently evaluated risk of bias by using the risk of bias tool suggested by the Cochrane Collaboration for programming and conducting systematic reviews and meta-analyses. The main outcome measure was sleep quality or the severity of insomnia, which was measured using subjective instruments, such as the Pittsburgh Sleep Quality Index (PSQI), Insomnia Severity Index (ISI), or objective instruments such as polysomnography, actigraphy, and safety of the intervention. For each outcome, a standardized mean difference (SMD) and confidence intervals (CIs) of 95% were determined.

**Results:**

Nineteen studies in this systematic review included 1832 participants. The meta-analysis of the combined data conducted according to Comprehensive Meta-Analysis showed a significant improvement in sleep (SMD = − 0.327, 95% CI = − 0.506 to − 0.148, *P* < 0.001). Meta-analyses revealed positive effects of yoga using PSQI scores in 16 randomized control trials (RCTs), compared with the control group in improving sleep quality among women using PSQI (SMD = − 0.54; 95% CI = − 0.89 to − 0.19; *P* = 0.003). However, three RCTs revealed no effects of yoga compared to the control group in reducing insomnia among women using ISI (SMD = − 0.13; 95% CI = − 0.74 to 0.48; *P* = 0.69). Seven RCTs revealed no evidence for effects of yoga compared with the control group in improving sleep quality for women with breast cancer using PSQI (SMD = − 0.15; 95% CI = − 0.31 to 0.01; *P* = 0.5). Four RCTs revealed no evidence for the effects of yoga compared with the control group in improving the sleep quality for peri/postmenopausal women using PSQI (SMD = − 0.31; 95% CI = − 0.95 to 0.33; *P* = 0.34). Yoga was not associated with any serious adverse events.

**Discussion:**

This systematic review and meta-analysis demonstrated that yoga intervention in women can be beneficial when compared to non-active control conditions in term of managing sleep problems. The moderator analyses suggest that participants in the non-breast cancer subgroup and participants in the non-peri/postmenopausal subgroup were associated with greater benefits, with a direct correlation of total class time with quality of sleep among other related benefits.

## Background

Sleep problems are one of the most common medical complaints. Lack of sleep is associated with significantly decreased work performance, impaired daytime function, and increased health care costs [1]. Sex-based differences in sleep problems have been widely published and discussed across sleep articles. Insomnia is approximately 1.41 times more common in women than in men [2]. Female populations at certain stages in their life span may be more vulnerable to insomnia. In these stages, hormonal changes associated with hormones, such as follicle-stimulating hormones (FSHs), luteinizing hormones (LHs), and progesterone, may play an important role in influencing women’s sleep construction [3] during adolescence [4], pregnancy and postpartum [5] or menopause [6]. Several behavioral, psychological and pharmacological treatments are available for insomnia, however, their efficacy varies considerably. The evidence of efficacy for cognitive behavior therapy is now well established in many reviews [7, 8], but availability remains poor. Pharmacotherapy remains the most common treatment [9], although hypnotics have been associated with many side effects, such as drowsiness, cognitive impairment, dependence, tolerance and poor long term efficacy [10].

Yoga has been widely adapted in the modern Eastern and Western hemispheres in a variety of ways. Yoga is an ancient form of exercise that focuses on strength, flexibility, and breathing to boost physical, mental and spiritual health [11]. There are many different styles of yoga, such as Tibetan, Iyengar, and Hatha Yoga. Some styles are more vigorous than others, whereas some may have different areas of emphasis, such as posture or breathing. The main components of yoga in Europe or America are mostly associated with physical posture (*Asana*) and breathing control (*Pranayama*) and meditation (*Dhyana*) [11]. A trial in yoga for persistent fatigue breast cancer survivors showed yoga is safe and effective at improving fatigue severity, depressive moods, and sleep quality [12]. Yoga is also characterized as a mindful mode of physical activity. Mindfulness, as an important component of yoga, improves sleep disturbance by increasing melatonin levels, reducing hyperarousal, and addressing stress related cardiac and respiratory abnormalities [13].

The term “sleep quality” is commonly used in sleep medicine. In 1989, Buysse et al. referred to sleep quality as a “complex phenomenon that is difficult to define and measure objectively” [14]. Good sleep quality is associated with better health, less daytime sleepiness, greater well-being and better psychological functioning [15]. Recently, sleep quality is defined as one’s satisfaction of the sleep experience, integrating aspects of sleep initiation, sleep maintenance, sleep quantity, and refreshment upon awakening [16]. The National Sleep Foundation (NSF) released the key indicators of good sleep quality, as established by a panel of experts. They include increase in sleeping time while in bed (at least 85% of the total time), falling asleep in 30 min or less, waking up no more than once per night and being awake for 20 min or less after initially falling asleep. However, there was less or no consensus regarding sleep architecture or nap-related variables as elements of good sleep quality [17]. Poor sleep quality is one of the defining features of chronic insomnia [18]. Although recent systematic reviews and meta-analyses have assessed the efficacy and safety of yoga in specific groups of women, such as those with prenatal depression [19] and primary dysmenorrhea [20] in different stages, evidence for the efficacy of yoga in improving sleep quality and insomnia of women has not yet been systematically assessed. Thus, the aim of this review was to systematically evaluate and perform a meta-analysis of the available data on the efficacy and safety of yoga in terms of improving sleep quality and insomnia in women.

## Methods

Before beginning the review, we followed the checklist for systematic reviews in concurrence with the preferred reporting items for systematic reviews and meta-analyses (PRISMA) guidelines [21] and suggestions by the Cochrane Collaboration for programming and conducting systematic reviews and meta-analyses [22].

### Eligibility criteria

#### Types of studies

Randomized controlled trials (RCTs), randomized crossover studies, and cluster randomized trials were all eligible for this meta-analysis. No restrictions in terms of language and countries were applied.

#### Types of participants

Studies that included women (aged ≥18 years) with sleep problems were eligible. No restrictions on the ethnicity and comorbidity of participants were applied.

#### Types of interventions

No restrictions regarding yoga type, form, structure, frequency, duration or length of intervention programs were applied. Studies on cointerventions that included yoga as a part of multicomponent interventions were excluded because it would be difficult to distinguish the effects of yoga from additional modalities. Studies in control interventions that compared yoga treatments with nontreatment, usual care, wait-lists, and education without active physical exercise programs were all eligible.

#### Types of outcome measures

*The primary outcome* of this study was sleep quality. To be included in this review, studies had to assess at least one of the sleep quality measures by using standardized instruments and provide outcomes both at the baseline and follow-up for primary outcomes. In particular, instruments in question include subjective measurements, such as the Pittsburgh Sleep Quality Index (PSQI) and Insomnia Severity Index (ISI), or objective measurements, such as polysomnography (PSG) and actigraphy. The PSQI score have been recommended as a reliable, valid and standardized instrument to measure and to identify quality of sleep. The widely employed Pittsburgh Sleep Quality Index (PSQI), provides a measure of global sleep quality, including sleep latency, sleep duration, habitual sleep efficiency, sleep disturbances, use of sleeping medication, and daytime dysfunction [14]. The seven components of the PSQI are standardized of areas routinely assessed sleep complaints with possible range of 0–21 points. A global PSQI score of 5 or higher provided a sensitive and specific measure for poor sleep quality [14]. The ISI score is a reliable and valid instrument to quantify perceived insomnia severity. A global ISI score of 8 or higher is indicative of some degree of insomnia, while moderate insomnia has a score of 15–21 and severe insomnia with a score of 22–28 [23]. PSG or actigraphy reports the most complete and precise information on the construction and distribution of sleep periods, such as total sleep time (TST), sleep efficiency (SE), and wake time after sleep onset (WASO) [24]. Sleep quality is also sometimes measured from PSG and actigraphy. Among these objective indices are measures such as sleep onset latency, total sleep time, wake time after sleep onset, sleep efficiency, and number of awakenings [25].

*Secondary outcomes*: The secondary outcome included in this study was the safety of the intervention, which was assessed as number of patients with adverse events (AEs), including serious adverse events or nonserious events. Serious adverse events referred to those events that caused death, life-threatening situations, hospitalization, disability or permanent damage, congenital anomaly/birth defect, or the need for medical or surgical intervention to prevent any of the aforementioned outcomes [26]. All other adverse events were regarded as nonserious.

### Search methods

The search strategy comprised four electronic databases from their inception through June 01, 2019: Medline/PubMed, ClinicalKey, ScienceDirect, Embase, PsycINFO, and the Cochrane Library. The literature search was constructed around search terms for “yoga,” “women,” and “sleep” and was adapted for each database as necessary. The complete search strategy for PubMed was as follows: (“yoga” OR “asana” OR “pranayama” OR “dhyana”) AND (“women” OR “female”) AND (“sleep” OR “sleep quality” OR “sleep disturbance” OR “insomnia”). Additional reference lists of identified original articles or reviews, the table of the contents of the *Journal of Yoga and Physical Therapy*, and *Journal of National Taiwan Sports University* were searched manually.

Retrieved articles were scanned independently to verify their eligibility, and the entire text was assessed by two reviewers. A conflict of reviewers’ opinions on inclusion or exclusion of any article was discussed with a third reviewer to reach a consensus.

### Data extraction and management

Two reviewers independently extracted data on design (e.g., article setting, author/year, country of studies, and sampling strategy), participants (e.g., age, body max index, clinical characteristics, comorbid condition, and overall sample size), interventions (e.g., yoga type, frequency of sessions per week, duration of yoga intervention, and total length of intervention time), control interventions (e.g., type, frequency, length, and duration), and outcomes (e.g., outcome measures with sleep quality and safety-related events). A conflict of reviewers’ opinions was discussed with a third reviewer until consensus was reached.

### Risk of bias in individual studies

Two reviewers independently assessed the risk of bias in each study. There were seven domains of assessment for the risk of bias include in the following: (1) random sequence generation, (2) allocation concealment, (3) blinding of participants and personnel, (4) blinding of outcome assessment, (5) incomplete outcome data, (6) selective reporting, and (7) other biases using the Cochrane Systematic Review Manual risk of bias assessment tool [22]. All domains were scored as low risk, high risk, or unclear risk of bias and assessed individually. A risk of bias table was completed for each included study. A conflict of reviewers’ opinions was discussed with a third reviewer until consensus was reached.

### Data assessment of overall effect size

A meta-analysis was conducted with Review Manager 5 software (Version 5.3, The Nordic Cochrane Centre, Copenhagen) and Comprehensive Meta-Analysis Software using a random effects model if at least two studies assessing this specific outcome were obtainable. For continuous outcomes, standardized mean differences (SMDs) with 95% confidence intervals (CIs) were calculated as the difference in means between groups divided by the pooled standard deviation. For studies that did not report data with standard deviations, we calculated these values from standard errors, confidence intervals, or *t*-values. If adequate information was available, we would plan to perform subgroup analysis. The *p* value of the summary effect < 0.05 were regarded as indicating statistical significance.

A negative SMD was provided a definition to display the beneficial effects of yoga intervention compared with the control intervention for sleep quality outcomes. Cohen’s categories were used to assess the significance of the overall effect size, with SMD = 0.2–0.5: small effect size; SMD = 0.5–0.8: medium effect size; and SMD > 0.8: large effect size [27].

### Assessment of heterogeneity

Statistical heterogeneity between studies was analyzed using the I^2^ statistics and the Cochrane chi-square. The variance between studies was measured using the tau-square (Tau^2^). The level of heterogeneity was classified as I^2^ = 0–24%: low heterogeneity; I^2^ = 25–49%: moderate heterogeneity; I^2^ = 50–74%: substantial heterogeneity; and I^2^ = 75–100%: considerable heterogeneity. Given the low power of this test when only few studies or studies with a low sample size are included in a meta-analysis, a *P* value of ≤0.1 for the chi-square test was regarded as indicating significant heterogeneity [22].

### Moderator analyses

Moderator and meta-regression analyses were further performed to identify possible reasons for interstudy heterogeneity. The subgroup analysis produced prespecified covariates, including outcome measurement tools, participant type, study quality, study region, participant age, intervention duration and study sample size.

### Risk of publication bias

Risk of publication bias was evaluated for each meta-analysis that included at least 10 studies. Funnel plots generated using Review Manager 5 software was estimated from individual studies against each study’s standard error. Publication bias was evaluated through visual analysis, in which roughly the symmetrical funnel plot signifies no evidence of high risk of publication bias [28]. Potential publication bias was evaluated using the Egger’s Intercept Test, with *p* values < 0.05 signifying significant bias.

## Results

### Literature search

**Fig. 1:**
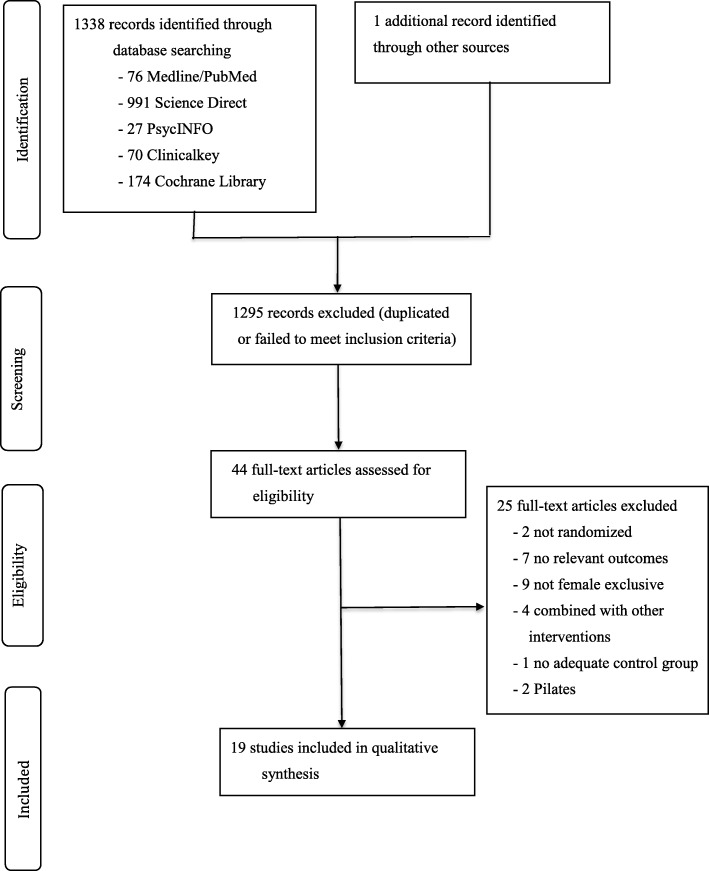
Flowchart of the results of the literature search

The results of the literature search and screening process are summarized in Fig. 1. The literature search totaled 1338 records; one additional record was retrieved from the Journal of National Taiwan Sports University in the Chinese language database [29]. In all, 1295 records were excluded because they did not meet all predefined inclusion criteria or were duplicated. Forty-four full-text articles were assessed for eligibility. Twenty-five were excluded because they were not randomized [29, 30], did not include relevant outcomes [31–37], did not include only female participants [38–46], included yoga as a part of a multimodal intervention (or combined with other intervention) [47–50], lacked adequate control [51], and did not include a form of yoga intervention [52, 53]. Nineteen full-text articles with 1832 participants were included in the qualitative synthesis and were included in the meta-analysis. All articles were published in English.

### Study characteristics

A total of 19 studies were considered eligible for systematic reviews. Information regarding the characteristics of the sample, yoga or control group interventions, outcome measures, and results are listed in Tables 1 and 2.

**Table 1:** Characteristics of included studies

Authors, year country	Main characteristics of the studied population	Sample characteristics (sample size, age)	Intervention group comparison group	Sleep outcome measures	Outcomes
Danhauer SC et al. 2009 [54] America	Women (≥18 y) with breast cancer (any stage), 2–24 months post-primary treatment (surgery) following initial diagnosis and/or had a recurrence of breast cancer within the past 24 months	44, G1 = 22, G2 = 22 Mean age: G1 = 54.3 y/o (SD = 9.6), G2 = 57.2 y/o (SD = 10.2)	G1 = Restorative Yoga G2 = Control group (wait list control)	PSQI	The total score of PSQI improved: No statistically significant finding G1 vs. G2 (*P* = 0.97)
Chandwani KD et al. 2010 [55] America	Women (≥18 y) with breast cancer (stage 0-stage III) who were scheduled to undergo radiotherapy at The University of Texas M.D. Anderson Cancer Center	61, G1 = 30, G2 = 31 Mean age: G1 = 51.39 y/o (SD = 7.97), G2 = 54.02 y/o (SD = 9.96)	G1 = Yoga G2 = Control group (wait list control)	PSQI	The total score of PSQI improved: No statistically significant finding G1 vs. G2 (*P* > 0.05)
Bower JE et al. 2012 [12] America	Post-menopausal women (aged 40–60 y) diagnosed with Stage 0 – II breast cancer; completed local and/or adjuvant cancer therapy (with the exception of hormonal therapy) at least 6 months and experiencing persistent cancer-related fatigue	31, G1 = 16, G2 = 15 Mean age: G1 = 54.4 y/o (SD = 5.7), G2 = 53.3 y/o (SD = 4.9)	G1 = Iyengar Yoga G2 = Control group (health education)	PSQI	The total score of PSQI improved: No statistically significant finding G1 vs. G2 (*P* > 0.05)
Kiecolt-Glaser JK et al. 2014 [56] America	Women (stage 0-IIIa breast cancer survivors in age from 27 to 76 y) had completed cancer treatment within the past 3 years (except for tamoxifen / aromatase inhibitors) and were at least 2 months post surgery or adjuvant therapy or radiation	200, G1 = 100, G2 = 100 Mean age: G1 = 51.8 y/o (SD = 9.8), G2 = 51.3y/o (SD = 8.7)	G1 = Hatha Yoga G2 = Control group (wait list control)	PSQI	The total score of PSQI improved: G1 vs. G2 (*P* = 0.03)
Ratcliff CG et al. 2016 [57] America	Women (aged ≥18 y) with breast cancer (diagnosed with stage 0 to III) scheduled to undergo daily adjuvant XRT (radiotherapy treatment) for 6 weeks at MD Anderson Cancer Center	163, G1 = 53, G2 = 56, G3 = 54 Mean age: G1 = 52.38 y/o (SD = 1.35), G2 = 51.14 y/o (SD = 1.32), G3 = 52.11 y/o (SD = 1.34)	G1 = Yoga G2 = Stretching G3 = Control group (wait list control)	PSQI	The total score of PSQI improved: No statistically significant finding G1 vs. G3 (*P* > 0.05)
Taylor T R et al. 2018 [71] America	Women (aged 18–65 y), no pregnant, breast cancer survivor of at least 12 months post-surgery and treatment (excluding hormone therapy), free of medical contraindications reported by their physician	33, G1 = 18, G2 = 15 Mean age: G1 = 54.9 y/o (SD = 8.8), G2 = 52.6 y/o (SD = 8.2)	G1 = Restorative yoga G2 = Control group (wait list control)	ISI	The total score of ISI improved: No statistically significant finding G1 vs. G2 (*P* = 0.89)
Chaoul A et al. 2018 [58] America	Women (aged ≥18 y) with breast cancer stage (American Joint Committee on Cancer (AJCC) TNM) I to III who were undergoing chemotherapy, were able to read, write and speak English; and were scheduled to undergo neoadjuvant or adjuvant therapy (weekly or every 21 days) at The University of Texas MD Anderson Cancer Center	227, G1 = 74, G2 = 68, G3 = 85 Mean age: G1 = 49.5 y/o (SD = 9.80), G2 = 50.4 y/o (SD = 10.3), G3 = 49.0 y/o (SD = 10.1)	G1 = Tibetan Yoga G2 = Stretching group G3 = Control group (Usual care)	PSQI Actigraphy	The total score of PSQI improved: No statistically significant finding G1 vs. G3 (*P* = 0.32) Actigraphy: Statistically significant finding in sleep efficiency (SE) G1 vs. G3 (*P* = 0.02), wake after sleep onset (WASO) G1 vs. G3 (*P* = 0.0003), but no statistically signify finding on sleep onset latency (OL) G1 vs. G3 (*P* = 0.89), total sleep time (TST) G1 vs. G3 (*P* = 0.19)
Porter LS et al. 2019 [59] America	Women (aged ≥18y) receiving treatment for metastatic breast cancer had a life expectancy ≥9 months as estimated by their treating oncologist; could speak and read English	63, G1 = 43, G2 = 20 Mean age: G1 = 56.3 y/o (SD = 11.6) G2 = 59.4 y/o (SD = 11.3)	G1 = Yoga G2 = Control group (social support group)	PSQI	The total score of PSQI improved: No statistically significant finding G1 vs. G2 (*P* > 0.05)
Elavsky S et al. 2007 [60] America	Sedentary or low-active middle-aged women (aged 42–58 y) during the menopausal transition who had no history of surgical menopause and had not used hormone therapy or at least 6 months. Baseline analyses revealed that overall sleep quality was poor in the sample (Mean PSQI = 6.21, SD = 3.46) with 88% of sample scoring	163, G1 = 61, G2 = 63, G3 = 39 Age range:42–58 y/oMean age:49.9 y/o (SD:3.6)	G1 = Yoga G2 = walking G3 = Control group	PSQI	The total score of PSQI improved: No statistically significant finding G1 vs. G3 (*P* > 0.05)
Afonso RF et al. 2012 [61] Brazil	Postmenopausal women (aged 50–65 y) with insomnia diagnosed by specialist based on DSM4, amenorrhea for 1 year or longer, had follicle-stimulating hormone (FSH) ≥ 30 mIU/ml, and had a BMI (Body mass index) < 30 kg/m^2^	61, G1 = 16, G2 = 21, G3 = 24 Age range:50–65 y/o	G1 = Yoga G2 = Passive stretching G3 = Control group	ISI Polysomnography	The total score of ISI improved: G1 vs. G3 (*P* < 0.05) Polysomnography: No statistically significant finding G1 vs. G3 (*P* > 0.05)
Newton KM et al. 2014 [62] America	Previous sedentary women (aged 40–62 y) in menopausal transition or postmenopausal or had hysterectomy with FSH ≥ 20 m IU/mL and estradiol ≤50 pg/mL, with ≥14 vasomotor symptoms /week in each of three consecutive weeks and had not used hormone therapy for past 1 month.	249, G1 = 107, G2 = 142 Age range:40–62 y/o	G1 = Yoga G2 = Control group (usual activity)	PSQI ISI	The total score of PSQI improved: G1 vs. G2 (*P* = 0.049) The total score of ISI improved: G1 vs. G2 (*P* = 0.007)
Buchanan, D.T. et al. 2017 [63] America	Women (aged 40–62 y) in menopausal transition or postmenopausal or had hysterectomy with FSH ≥ 20 mIU/mL and estradiol ≤50 pg/mL, generally in good health; experiencing 14 or more hot flashes/night sweats per week (on 2-w screening diaries); and hot flashes rated as bothersome or severe on four or more occasions/week	186, G1 = 52, G2 = 54, G3 = 80 Mean age: G1 = 55.3 y/o (SD = 3.9), G2 = 55.6 y/o (SD = 3.5), G3 = 54.2 y/o (SD = 3.7)	G1 = Yoga G2 = Exercise G3 = Control group (usual activity)	Actigraphy	Statistically no significant finding in sleep efficiency (SE) G1 vs. G3 (*P* > 0.05), wake after sleep onset (WASO) G1 vs. G3 (*P* > 0.05), sleep onset latency G1 vs. G3 (*P* > 0.05), total sleep time (TST) G1 vs. G3 (*P* > 0.05)
Ide MR et al. 2008 [64] Brazil	Women with fibromyalgia syndrome (1990 American College of Rheumatology criteria) with time availability	40, G1 = 20, G2 = 20 Mean age: G1 = 46.61 y/o (SD = 9.80), G2 = 45.47 y/o (SD = 8.65)	G1 = Yoga breathing exercises in warm water G2 = Control group	PSQI	The total score of PSQI improved: G1 vs. G2 (*P* = 0.004)
Innes KE et al. 2012 [65] America	Nonsmoking women (aged 45–79 y), post-menopausal (≥12 months amenorrheic) physical inactive (exercising less than 20 min, 3 times per week) and overweight (BMI ≥ 25 kg/m^2^ and/or waist circumference ≥ 88 cm) with restless legs syndrome	20, G1 = 10, G2 = 10 Mean age: G1 = 58.4 y/o (SD = 6.32), G2 = 58.9 y/o (SD = 9.10)	G1 = Iyengar Yoga G2 = Control group (education film group)	PSQI	The total score of PSQI improved: G1 vs. G2 (*P* = 0.01)
Cheung C et al. 2014 [66] America	Community-dwelling women (aged 65–90 y) had symptomatic osteoarthritis (OA) of knee diagnosis for at least 6 months without previous training in any form of yoga	36, G1 = 18, G2 = 18 Mean age: G1 = 71.9 y/o, G2 = 71.9 y/o	G1 = Yoga G2 = Control group (wait list control)	PSQI	The total score of PSQI improved: No statistically significant finding G1 vs. G2 (*P* = 0.15)
Fang R et al. 2015 [67] China	Female nurses (aged of 25–51 y) with normal communication abilities and willingness to participate study	120, G1 = 61, G2 = 59 Mean age: G1 = 35.13 y/o (SD =10.98), G2 = 36.05 y/o (SD = 9.91)	G1 = Yoga G2 = Control group	PSQI	The total score of PSQI improved: G1 vs. G2 (*P* < 0.001)
Ebrahimi M et al. 2017 [68] Iran	Women (aged 38–53 y) with Type 2 Diabetes mellitus lack of any diabetic complications, no participation in any kind of regular aerobic exercise and resistance training over the last 6 months, BMI < 40 kg/m^2^, not being under insulin treatment	45, G1 = 15, G2 = 15, G3 = 15 Mean age: G1 = 48.18 y/o, G2 = 44.69 y/o, G3 = 47.93 y/o	G1 = Yoga G2 = Aerobic exercise G3 = Control group	PSQI	The total score of PSQI improved: G1 vs. G3 (*P* < 0.05)
Rao M et al. 2017 [69] India	Female teachers, aged between 30 and 55 years were willing to participate in the study and had no previous exposure to any form of yoga practice.	60, G1 = 30, G2 = 30 Mean age: G1 = 43.0 y/o (SD = 9.77) G2 = 40.0 y/o (SD = 7.32)	G1 = Yoga-based, mindfulness relaxation G2 = Control group (wait list control)	PSQI	The total score of PSQI improved: G1 vs. G2 (*P* < 0.01)
Nalgirkar SP et al. 2018 [70] India	Women (aged 20–50 y) and diagnosed for primary dysfunctional uterine bleeding (DUB) with no underlying systemic pathology	30, G1 = 15, G2 = 15 Mean age: G1 = 29.85 y/o (SD =4.45) G2 = 30.85 y/o (SD =4.42)	G1 = Yoga G2 = Control group (wait list control)	PSQI	The total score of PSQI improved: No statistically significant finding G1 vs. G2 (*P* > 0.05)

*BMI* Body max index, *DSM4* Diagnostic and Statistical Manual of Mental Disorders, Fourth Edition criteria, *DUB* Dysfunctional uterine dysfunction, *ECOG-PS* Eastern Cooperative Oncology Group Performance Status, *FSH* Follicle-stimulating hormone, *G1* Group 1, *G2* Group 2, *G3* Group 3, *ISI* Insomnia Severity Index, *OA* Osteoarthritis, *OL* Onset latency, *PSQI* Pittsburgh Sleep Quality Index, *SE* Sleep efficiency, *TST* Total sleep time, *WASO* Wake time after sleep onset, *XRT* Radiotherapy treatment

**Table 2:** Characteristics of yoga programs and outcome assessment of studies included in the systematic review

Authors, year country	Specific type of yoga	Yoga frequency (sessions/week)	Session length (mins/week)	Study duration (weeks/ study)	Number of sessions/study Total lengths (h)/study	Safety (adverse events)	Basal score of PSQI (SD) and follow-up	Basal score of ISI (SD) and follow-up
Elavsky s et al. 2007 [60] America	Iyengar Yoga (Hatha Yoga)	2	90	16	32 (24 h)	Not reported	G1 = 6.9 0(3.94) G1 = 6.48 (4.22) G3 = 5.46 (2.96) G3 = 5.44 (3.63)	–
Afonso RF et al. 2012 [61] Brazil	Asanas Yoga	2	120	16	32 (32 h)	Not reported	–	G1 = 14.1 (5.87) G1 = 9.7 (4.64) G3 = 15.2 (4.8) G3 = 13.7 (4.64)
Newton KM et al. 2014 [62] America	Yoga program	2	90	12	24 (18 h)	Reported	G1 = 7.7 (3.34) G1 = 5.6 (3.30) G2 = 8.4 (3.30) G2 = 5.8 (2.91)	G1 = 11.8 (5.25) G1 = 7.4 (5.07) G2 = 12.2 (5.13) G2 = 6.8 (4.35)
Buchanan, D.T. et al. 2017 [63] America	Viniyoga	1	90	12	12 (18 h)	Not reported	–	–
Danhauer SC et al. 2009 [54] America	Restorative Yoga	1	75	10	10 (12.5 h)	Not reported	G1 = 8.3 (4.7) G1 = 6.1 (4.3) G2 = 8.6 (5.3) G2 = 7.0 (4.2)	–
Chandwani KD et al. 2010 [55] America	Yoga	2	120	6	12 (12 h)	Not reported	G1 = 7.3 (3.83) G1 = 7.3 (4.67) G2 = 7.1 (3.89) G2 = 7.1 (5.38)	–
Bower JE et al. 2012 [12] America	Iyengar Yoga	2	90	12	24 (36 h)	Not reported	G1 = 9.2 (3.3) G1 = 8.1 (2.5) G2 = 9.1 (3.5) G2 = 7.7 (2.6)	–
Kiecolt-Glaser KJ et al. 2014 [56] America	Hatha Yoga	2	180	12	24 (36 h)	Reported	G1 = − G1 = 7.0 (2.15) G2 = − G2 = 6.3 (2.18)	–
Cheung C et al. 2014 [66] America	Hatha Yoga	1	60	8	8 (8 h)	Not reported	G1 = 6.5 (4.2) G1 = 5.0 (2.2) G2 = 5.4 (2.8) G2 = 6.1 (2.2)	–
Ratcliff CG et al. 2016 [57] America	Yoga program	3	180	6	18 (18 h)	Not reported	G1 = 8.3 (3.9) G1 = 6.7 (3.1) G3 = 8.2 (3.7) G3 = 7.3 (3.7)	–
Taylor TR et al. 2018 [71] America	Restorative Yoga	1	75	8	8 (10 h)	Not reported	–	G1 = 10.18 (8.74) G1 = 7.89 (7.17) G2 = 7.56 (6.82) G2 = 6.20 (7.11)
Chaoul A et al. 2018 [58] America	Tibetan Yoga	4	300–360	1	4 (5–6 h)	Not reported	G1 = 7.8 (3.7) G1 = 7.3 (3.6) G3 = 8.1 (4.2) G3 = 8.1 (4.4)	–
Porter LS et al. 2019 [59] America	Mindful Yoga	1	120	8	8 (16 h)	Not reported	G1 = 8.6 (3.34) G1 = 8.6 (3.01) G2 = 7.6 (2.73) G2 = 7.6 (3.42)	–
Ide MR et al. 2008 [64] Brazil	Yoga breathing exercises in warm water	4	240	4	16 (16 h)	Not reported	G1 = 13.17 (4.00) G1 = 9.95 (1.15) G2 = 11.82 (5.05) G2 = 13.88 (1.28)	–
Innes K E et al. 2012 [65] America	Iyengar yoga	2	180	8	16 (24 h)	Not reported	G1 = 8.71 (3.63) G1 = 3.57 (1.49) G2 = 9.25 (3.32) G2 = 8.00 (2.94)	–
Fang R et al. 2015 [67] China	Yoga	> 2	> 100–120	24	> 48 (40–48 h)	Not reported	G1 = 9.98 (1.89) G1 = 7.61 (1.25) G2 = 10.24 (2.35) G2 = 10.31 (2.42)	–
Ebrahimi M et al. 2017 [68] Iran	Yoga program	3	270	12	36 (54 h)	Not reported	G1 = 14.40 (5.92) G1 = 3.73 (3.49) G3 = 13.91 (5.52) G3 = 13.27 (5.58)	–
Rao, M et al. 2017 [69] India	Yoga-based, mindfulness relaxation	5	150	4	20 (10 h)	Not reported	G1 = 5.63 (3.31) G1 = 3.10 (1.26) G2 = 4.86 (2.52) G2 = 5.9 (1.93)	–
Nalgirkar SP et al. 2018 [70] India	Yoga program	3	180	12	36 (h)	Not reported	G1 = 15.16 (8.29) G1 = 12.75 (4.73) G2 = 9.91 (4.69) G2 = 10.08 (3.75)	–

#### Study and participant characteristics

Of the 19 RCTs that were included in Table 1, six RCTs included healthy participants [60–63, 67, 69], including nurses [67], teachers [69], and women in the menopausal transition period or postmenopausal period [60–63]. The other 13 RCTs included breast cancer patients undergoing treatment [55, 57–59], breast cancer patients who had completed treatment [12, 54, 56, 71], type 2 diabetes mellitus patients [68], fibromyalgia patients [64], knee osteoarthritis patients [66], restless leg syndrome patients [65], and patients experiencing dysfunctional uterine bleeding [70].

Overall, the 19 RCTs included were conducted in the United States [12, 54–60, 62, 63, 65, 66, 71], Brazil [61, 64], India [69, 70], Iran [68], and China [67]. Study participants were recruited from hospitals [54, 55, 57, 58, 67, 68, 70, 71], outpatient clinics [59, 61] and schools [69]. The process of recruitment also included using purchased lists and health-plan enrollment files [62, 63] and multiple other mechanisms, including flyers, newspaper advertisements, web-based announcements, brochures, public health departments, tumor registry systems, and doctor referrals [12, 56, 60, 65, 66]. One study did not reveal the source from which participants were recruited [64]. Nineteen studies included in the systematic review displayed a baseline of PSQI higher than 5 or ISI higher than 8, indicating poor sleep quality or insomnia. The only exceptions were two studies, with individual control groups in each study displaying a baseline of PSQI lower than 5 [69] or ISI lower than 8 [71]. The sample size ranged from 20 to 249 with a median of 96. Participant’s mean age ranged from 29.8 to 71.9 years, with a median of 50.1 years. All participants were women.

#### Intervention characteristics

Of the 19 included studies in Table 1, three reported using Iyengar Yoga [12, 60, 65]; two reported using Hatha Yoga [56, 66]; two reported using Tibetan Yoga [58, 61]; two reported using Restorative Yoga [54, 71]; one reported using Vini Yoga [63]; one reported using Yoga Relaxation with MindSound Resonance Technique [69]; one reported using yoga breathing exercise in warm water [64]; and only seven RCTs revealed yoga programs with postures, breathing, relaxation or mediation, without defining a specific style of yoga [55, 57, 59, 62, 67, 68, 70]. All RCTs included yoga postures in their yoga intervention; 16 RCTs included yoga breathing; 15 RCTs included yoga relaxation; 12 RCTs included meditation; and 7 RCTs included all contents with postures, breathing, relaxation, and meditation for the yoga intervention group [55, 57, 62, 67, 68, 70, 71]. The duration of yoga interventions ranged from 1 week to 24 weeks, with a median of 10 weeks; the frequency of yoga interventions ranged from one to five weekly sessions of 45 to 120 min. Sixteen studies compared the yoga group with waitlist control groups with no specific treatment; three studies compared the yoga group with the control group, including two studies for education groups [12, 65] and one study for social support groups [59].

#### Outcome measures

All studies evaluated outcomes directly at the end of interventions. All studies assessed the subjective or objective measurements of sleep quality: 16 RCTs used the PSQI; three RCTs used the ISI [61, 62, 71]; one RCT used PSG [61]; and two RCTs used actigraphy [58, 63]. Safety-related events were reported in only two RCTs [56, 62].

### Risk of bias

#### Risk of bias in individual assessments

**Fig. 2:**
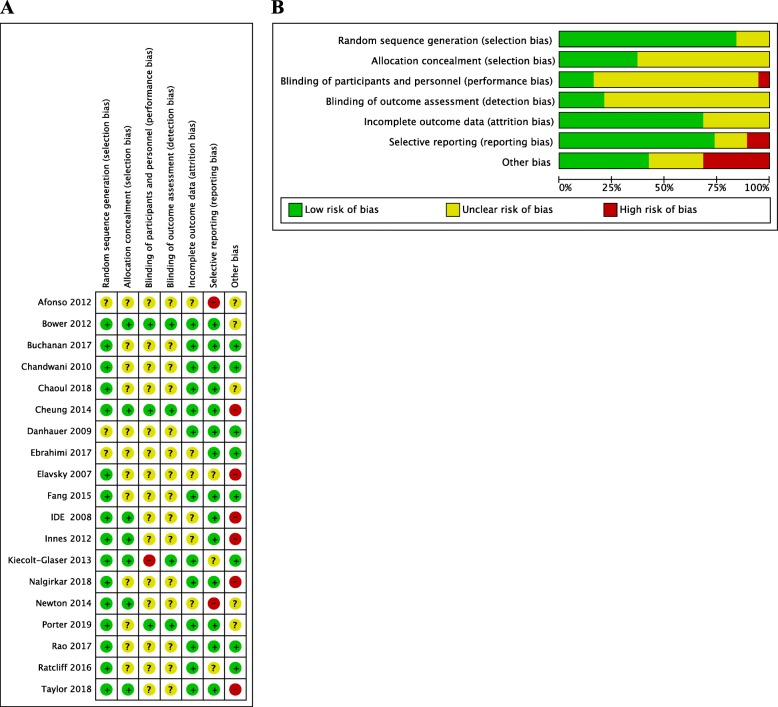
Risk of bias in individual studies. +, low risk of bias;?, unclear risk of bias; −, high risk of bias (a). Risk of bias for each criterion presented as percentages across all included studies (b)

Graphical representation of the risk-of-bias assessment is represented in Fig. 2. All studies had a high or unclear risk of bias in at least one domain. All studies claimed to be randomized; however, three studies did not reveal their content and method of random sequence [54, 61, 68]. Twelve studies did not report methods applied to perform adequate allocation [54, 55, 57–61, 63, 67–70]. Most studies offered no data material on blinding. Three studies clearly reported that participants and personnel were blinded [12, 59, 66]. Four studies clearly reported that researchers and outcome assessments were blinded [12, 56, 59, 66]. Six studies had insufficient data on attrition rates [60–62, 64, 65, 68]. Twelve studies had a low risk of selection reporting; only two studies had a high risk of selective reporting due to several reported outcome parameters not being revealed in study protocol or duplicate publications reporting different results of the same trial [61, 62]. Six studies had a high risk of other potential sources of bias due to poor participant compliance, intervention length, sample size or baseline differences [60, 64–66, 70, 71].

#### Publication bias

**Fig. 3:**
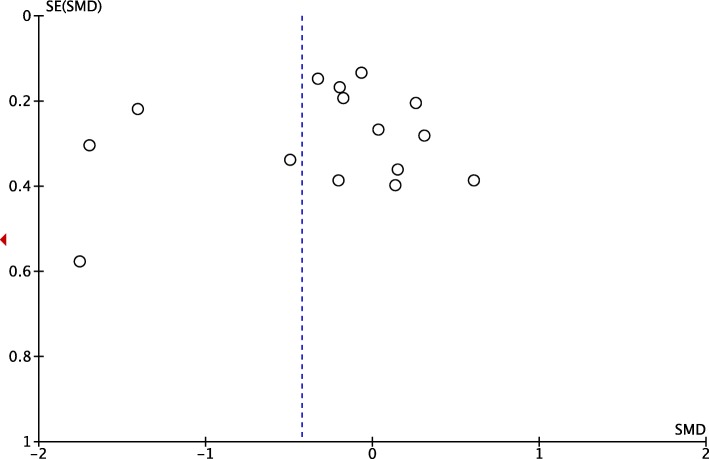
Funnel plot of estimate of publication bias in meta-analysis of the effects of yoga on women’s sleep quality compared to control groups (PSQI). SE: standard error; SMD: standardized mean difference

The meta-analysis of the effect of yoga on the sleep quality of women that involved yoga groups compared with control groups included 16 studies. The asymmetrical shape of the funnel plot indicated that subjective publication bias was detected (Fig. 3). Objective publication bias was analyzed using Egger’s Regression Test. Egger’s Test consists of the regression between the accuracy of the studies and standardized effects, which are weighted by the inverse of variance. Borderline findings (*P* = 0.05) show objective evidence on publication bias between precision and standardized effects of studies in the present study, specifically suggesting need for future studies to expound on the issue.

### Analysis of overall effects

#### Primary outcomes

The random effects model was applied to analyze the 19 RCTs outcomes by different sleep outcome measurement tools. The meta-analysis of combined data conducted with Comprehensive Meta-Analysis, showed a significant improvement in sleep problems (SMD = -0.327, 95% CI = − 0.506 to − 0.148, *P* < 0.001). However, significant heterogeneity existed among all the studies (Q = 43.152, I^2^ = 58.287%, *P* = 0.001). Therefore, moderator and meta-regression analyses were conducted to further explore the determinants of the heterogeneity.

**Fig. 4:**
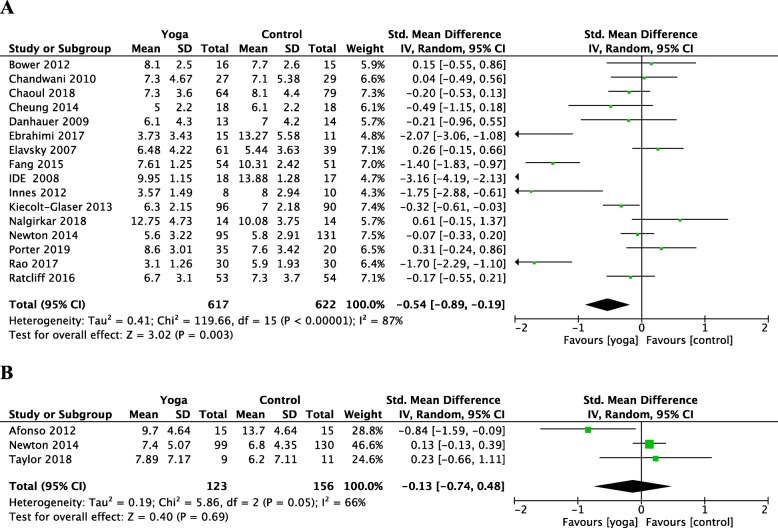
Forest plots for the effects of yoga on sleep quality in women versus control groups. **a** the global score of the Pittsburgh Sleep Quality Index (PSQI) **b** the global score of the Insomnia Severity Index (ISI). CI, confidence interval; IV, inverse variance; SD, standard deviation

The meta-analysis revealed the effects of yoga compared with the control group on the sleep quality and insomnia of women using the PSQI or ISI, as displayed in Fig. 4. Sixteen RCTs revealed evidence for effects of yoga compared with the control group in improving sleep quality in women using the PSQI (SMD = − 0.54; 95% CI = − 0.89 to − 0.19; *P* = 0.003). However, three RCTs revealed no effects of yoga compared with the control group in reducing the severity of insomnia in women using ISI (SMD = − 0.13; 95% CI = − 0.74 to 0.48; *P* = 0.69). Two RCTs revealed no effects of yoga compared with control group in improving sleep efficiency (SMD = 0.85; 95% CI = − 0.56 to 2.26; *P* = 0.26) or total sleep time (SMD = − 0.06; 95% CI = − 0.26 to 0.13; *P* = − 0.59) in women using actigraphy.

#### Secondary outcomes (safety)

Only two studies reported safety-related events. Two events revealed in one study could potentially be attributed to yoga intervention: two women reported the recurrence of chronic back and/or shoulder problems [56]. In another study, adverse events reported did not differ between the yoga intervention group and the control group (*P* = 0.41). These adverse events included muscle aches and strains (6.7%, yoga group; 10.3%, control group), low back pain (4.2%, yoga group; 3.1%, control group), or changes in strength or sensation in arms and legs (5.5% yoga group; 8.9% control group). Dropouts were not regarded as being adverse events because they did not explicitly show a possible reason or explanation for dropout in the original study. No serious adverse effects were reported in the included studies.

#### Subgroup analyses

**Fig. 5:**
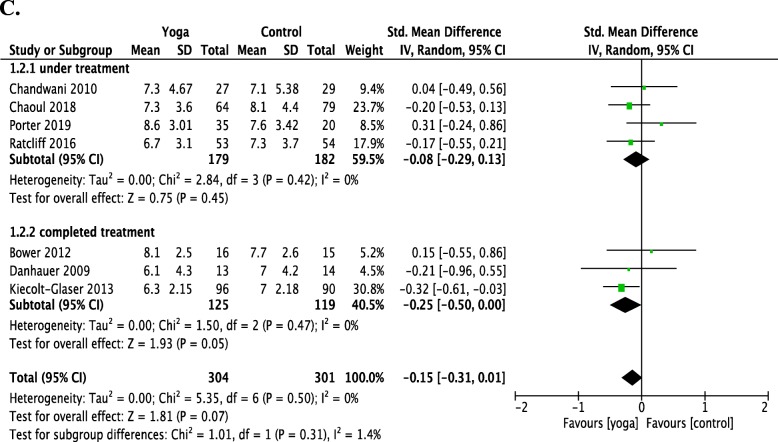
**c** Forest plots of the effects of yoga on the sleep quality of women with breast cancer (including women under treatment and women who had completed treatment) versus a control group using the global score of the Pittsburgh Sleep Quality Index (PSQI). CI, confidence interval; IV, inverse variance; SD, standard deviation

Participants were divided into two separate subgroups. Meta-analyses revealed the effects of yoga compared with the control group for women with breast cancer in Fig. 5. Seven RCTs revealed no evidence for the effect of yoga compared with the control group in improving sleep quality for women with breast cancer using the PSQI (SMD = − 0.15; 95% CI = − 0.31 to 0.01; *P* = 0.5). Four RCTs revealed no evidence for effects of yoga compared with the control group in improving sleep quality for women undergoing treatment for breast cancer (SMD = − 0.08; 95% CI = − 0.29 to 0.13; *P* = 0.45). Three RCTs revealed no evidence for positive effects of yoga in terms of improving sleep quality for women with breast cancer who had completed treatment compared with the control group (SMD = − 0.25; 95% CI = − 0.50 to 0.00; *P* = 0.05).

**Fig. 6:**
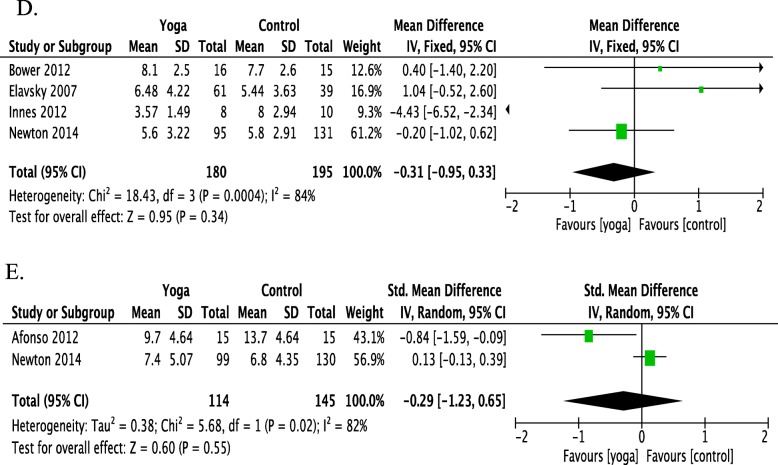
Forest plots displaying the effects of yoga versus control groups on sleep quality in peri/postmenopausal women in (**d**) the global score of the Pittsburgh Sleep Quality Index (PSQI) (**e**) the global score of Insomnia Severity Index (ISI). CI, confidence interval; IV, inverse variance; SD, standard deviation

The meta-analysis showed evidence of the positive effects of yoga on sleep quality compared with control groups for peri/postmenopausal women as displayed in Fig. 6. Four RCTs revealed no evidence for effects of yoga compared with control groups in improving sleep quality in peri/postmenopausal women using the PSQI (SMD = − 0.31; 95% CI = − 0.95 to 0.33; *P* = 0.34). Two RCTs revealed no evidence for effects of yoga compared with the control group in reducing severity of insomnia in peri/postmenopausal using ISI (SMD = − 0.29; 95% CI = − 1.23 to 0.65; *P* = 0.55).

### Moderator analyses, meta-regression

Moderator analyses and meta-regression are presented in Table 3. Significant factors in observed heterogeneity were identified in yoga on sleep quality and insomnia in women with sleep problems. Studies that used PSQI as outcome measurement tool showed a greater reduction in sleep problems than other studies that used other instruments as outcome measurement tools (*Hedges*’ g = − 0.369 vs. 0.031, *P* = 0.002). Participants without breast cancer showed more improvement in sleep problems than participants with breast cancer (*Hedges*’ g = − 0.522 vs. -0.148, *P* = 0.001). Studies without peri/postmenopausal women showed more improvement in sleep problems than studies with peri/postmenopausal women (*Hedges*’ g = − 0.419 vs. -0.084, *P* = 0.003). Regression analyses revealed a positive correlation with total length of class hours (*p* = 0.003), indicating that more total class hours, increased the chance to have significant results. Regression analyses revealed a negative correlation with mean age (*p* = 0.003) and sample size (*p* = 0.032) of study, indicating that the younger, and smaller sample sizes were more likely to have significant results.

**Table 3:** Mean effect sizes and moderator analyses of yoga in women with sleep problems

Parameter	Results	Effect Size (Hedges’g)	95%CI	*P*
**Categorical Moderators**				
**Outcome measurement tool**				
PSQI	16	- 0.369	−0.559, − 0.178	0.002^*^
Others	3	0.031	− 0.265, 0.328	
**Participant**				
Breast cancer group	8	−0.148	−0.304, 0.009	0.001^*^
Non-breast cancer group	11	−0.522	−0.821,-0.224	
**Participant**				
Peri/postmenopausal	6	−0.084	−0.269, 0.102	0.003^*^
Others	13	−0.419	−0.647,-0.191	
**Study region**				
American	13	−0.123	−0.240,-0.006	< 0.001^*^
Others	6	−0.844	−1.114,-0.573	
**Random sequence generation**				
High/ unclear risk	3	−0.578	−1.272, 0.116	0.001^*^
Low risk	16	−0.292	−0.473,-0.111	
**Continuous Moderators**				
**Sample size**	19		0.000,0.003	0.032^*^
**Total length of class time**	19		−0.021,-0.004	0.003^*^
**Study duration**	19		−0.03,0.087	0.20
**Mean age**	17		0.008,0.035	0.003^*^

*P value <0.05 indicated a significant difference

### Sensitivity analyses

In the included studies with low risk of selection bias, reporting bias, and other bias, the effect of yoga group compared to control group on women sleep PSQI did not change substantially, including random sequence generation bias (SMD = − 0.45; 95% CI = − 0.84 to − 0.11; *P* = 0.01; heterogeneity: I^2^ = 88%; χ^2^ = 107.43, *P* < 0.00001), allocation concealment bias (SMD = − 0.77; 95% CI = − 1.37 to − 0.16; *P* = 0.01; heterogeneity: I^2^ = 88%; χ^2^ = 40.95, *P* < 0.00001), selective reporting bias (standard mean difference = − 0.59; 95% CI = − 1.10 to − 0.08; *P* = 0.02; heterogeneity: I^2^ = 88%; χ^2^ = 93.11, *P* < 0.00001) and other bias (standard mean difference = − 0.53; 95% CI = − 1.03 to − 0.04; *P* = 0.03; heterogeneity: I^2^ = 86%; χ^2^ = 44.03, *P* < 0.00001). The effect compared with the control group remained significant in terms of sensitivity analyses of performance bias, detection bias, or attrition bias after eliminating high risk bias or uncertain risk bias of the studies.

## Discussion

### Summary of evidence

In this systematic review of 19 studies for yoga’s effect on improving women’s sleep quality and severity of insomnia, 19 RCTs revealed evidence for yoga improving sleep problems in women (SMD = − 0.327, 95% CI = − 0.506 to − 0.148, *P* < 0.001). As shown in Fig. 4, 16 RCTs meta-analysis suggests yoga can bring 1.2 points improvement in PSQI score (SMD = − 0.54; 95% CI = − 0.89 to − 0.19; *P* = 0.003). However, seven RCTs revealed no evidence for yoga improving sleep quality in women with breast cancer (Fig. 5, SMD = − 0.15; 95% CI = − 0.31 to 0.01; *P* = 0.5). Four RCTs revealed no evidence for improving PSQI in peri/postmenopausal women (Fig. 6, SMD = − 0.31; 95% CI = − 0.95 to 0.33; *P* = 0.34). Two RCTs revealed no evidence for improving ISI in peri/postmenopausal women (Fig. 6, SMD = − 0.29; 95% CI = − 1.23 to 0.65; *P* = 0.55).

However, heterogeneity of effects were high across all studies. In Table 3, our moderator analyses yielded statistically significant differences, the effect of yoga for improving sleep problems in non-breast cancer subgroup, non peri/postmenopausal subgroup are superior to breast cancer subgroup, peri/postmenopausal subgroup.

Overall, the application of yoga was not associated with worsening of sleep problems or increased adverse effects. Only two studies explicitly assessed safety-related nonserious adverse events. Yoga is most likely a comparatively safe intervention in this population. However, future RCTs should take more measures to ensure stricter reporting of adverse events and reasons for dropouts.

### Comparison with prior reviews

There was no systematic review available that explicitly focused on yoga for improving sleep quality and insomnia in a specific gender. Ours is the first systematic review and meta-analysis with 19 RCTs that to focus on the effects of yoga on women with sleep problems. A previous review published until February 2019 included subgroup analysis of yoga on mind-body therapies on insomnia [72]. This recent review illustrated that yoga had beneficial effects on subjective sleep quality in participants in all gender groups. Our meta-analysis with 16 RCTs uncovered evidence for the effects of yoga on the sleep quality in women. Only six RCTs were found to have overlapped with this previous review [58, 61, 62, 65–67]. Our meta-analysis also examined the potential effect on specific subgroups, such as breast cancer and peri/postmenopausal subgroups, with these subgroups serving as potential factors in sleep quality effects (although the result did not show any clear difference). Significant subgroup differences were identified for the following participants types: (peri/postmenopausal vs. non peri/postmenopausal, breast cancer vs. non-breast cancer). Results from the peri/postmenopausal subgroup of women in our systematic review also agreed with previous published reports that suggested that yoga had no significant effect on the severity of insomnia in middle-aged women [73]. There were baseline differences between participants based on intervention assignment in PSQI scores [62, 65]. This may have contributed to results displaying no significant effect in sleep quality in the peri/postmenopausal subgroup of women. Yoga seems to be effective for reducing total menopausal symptoms including psychological, somatic, vasomotor and in previous systematic review and meta-analysis [74], but there is no direct answer in the study focusing on reducing sleep problems. Future research should ensure more rigorous methodology and adequate sample size concerning the effects of yoga on quality of sleep improvement among the subgroup of peri/postmenopausal women.

Compared to yoga intervention, previous systematic reviews also indicate that programmed exercise improved sleep quality in middle-aged women [73]. However, these reviews are also limited to high heterogeneity of clinical evidence and failed to provide any specific suggestions for exercise dosages or formats. Additionally, other reviews included an overly wide range of nonpharmacological interventions ranging from walking [75], tai chi [76], qigong exercise [72] showing evidence of beneficial effect in improving self-rated sleep quality. However, despite this, heterogeneity remained high due to difference of interventions and target populations. Our meta-analysis conducted to further explore the determinants of the heterogeneity with subgroup analysis for categorical moderators and continuous moderators to find significant factors for observed heterogeneity.

### External and internal validity

Major threats to external validity included the specificity of variables of sampled participants and multiple yoga types or styles. The majority of RCTs included participants from North America, South America, and Asia; lacking studies from Europe and Africa. It might not be as universally transferable to other areas.

There were several other limitations in this review: the wide variety of diagnoses included; the inclusion of only certain types of people or professions, such as nurses, teachers, and peri/postmenopausal women; and patients with breast cancer, type 2 diabetes mellitus, fibromyalgia syndrome, osteoarthritis of the knee, restless leg syndrome, and primary dysfunctional uterine bleeding. The heterogeneity of interventions with different types or styles of yoga (postures, breathing, relaxation, or mediation), and potential bias were included in this systematic review.

Other threat to internal validity was study bias. Only few effects were robust against all potential bias. All of our studies claimed to have applied randomization methods; however, three RCTs failed to provide the design protocol of randomization. Some of the included studies may not have been truly randomized. Erroneous allocation concealment has been empirically revealed to be a significant source of bias in RCTs [77]. Our included studies only had a low risk or an unclear risk of detection bias without high risk detection bias. The results of meta-analysis did no changed when studies excluded high risk or unclear risk reviews on selection bias or reporting bias. The internal validity of the review appeared to be limited but acceptable.

### Strengths and weaknesses

This is the first and latest systematic review and meta-analysis available on yoga for sleep quality and insomnia in women. A large number of RCTs on female population-related physiological and physiological comorbidities and risk factors in insomnia were included. There were five primary limitations of this review. First, subjective publication bias revealed in this review may have been due to selective reporting bias, which means that articles with negative findings may have not been published or poor methodological quality of including articles. We have applied Egger’s Test for objective publication bias in our review. Second, the participant characteristics included in the review were heterogeneous; subgroups were included to analyze the effectiveness of different participant groups; and the small number of RCTs limited data presentation. Third, the severity of the sleep complaints and health status of participants was not considered or individually listed in each study. Baseline differences in PSQI scores were found between intervention and control groups in three studies [56, 62, 65]. This may have led to heterogeneity. The fourth limitation was the intensity, frequency, and duration of yoga interventions were heterogeneous; short term applications of less than 1 month yoga intervention were found in some studies [58, 64, 69]. Only four reviews reported long-term follow up effects, ranging from 3 months to 12 months [55–58]. Lastly, lack of safety issue evaluation including serious adverse events or nonserious events in each study.

### Implications for further research

This systematic review and meta-analysis was limited by the low methodological quality of included studies. Further RCTs should ensure rigorous methodology and reporting, which would mean adequate sample size, adequate randomization, allocation concealment, intention-to-treat analysis, and blinding of at least outcome assessors [78]. Researchers for study interventions may need to apply a standard protocol. Adequate reporting of safety issues with yoga intervention should be discussed in future randomized controlled trials. Evidence was limited because few studies report safety-related adverse effects. Most of the included studies failed to report this aspect.

## Conclusion

This systematic review and meta-analysis demonstrated that yoga intervention in women has benefits compared to non-active control conditions in term of managing sleep problems. The moderator analyses suggested that participants in the non-breast cancer subgroup or participants in the non-peri/postmenopausal subgroup were associated with greater benefits, with the longer total length of class time, the more beneficial these practices were.

## Data Availability

All data analyzed during this study are included in this published article and the original studies’ publications.

## Abbreviations

AEs: Adverse events
BMI: Body max index
CI: Confidence interval
DSM4: Diagnostic and Statistical Manual of Mental Disorders, Fourth Edition criteria
DUB: Dysfunctional uterine dysfunction
ECOG-PS: Eastern Cooperative Oncology Group Performance Status
FSH: Follicle-stimulating hormone
G1: Group 1
G2: Group 2
G3: Group 3
ISI: Insomnia Severity Index
IV: Inverse variance
LH: Luteinizing hormones
MD: Mean differences
OA: Osteoarthritis
OL: Onset latency
PRISMA: Preferred reporting items for systematic reviews and meta- analyses
PSQI: Pittsburgh Sleep Quality Index
RCT: Randomized controlled trial
SD: Standard deviation
SE: Sleep efficiency
SE: Standard error
SMD: Standardized mean differences
SWS: Slow- wave sleep
TST: Total sleep time
WASO: Wake time after sleep onset
XRT: Radiotherapy treatment
